# Clinical case of 45,X/46,XY mosaic male with ejaculatory disorder associated with seminal vesicle dysplasia: a case report

**DOI:** 10.1093/sexmed/qfae066

**Published:** 2024-10-01

**Authors:** Jurii Karibe, Teppei Takeshima, Daiji Takamoto, Takashi Kawahara, Kimito Osaka, Jun-ichi Teranishi, Kazuhide Makiyama, Hiroji Uemura, Yasushi Yumura

**Affiliations:** Department of Urology, Reproduction Center, Yokohama City University Medical Center, Yokohama, 232-0024, Japan; Department of Urology and Renal Transplantation, Yokohama City University Medical Center, Yokohama, 232-0024, Japan; Department of Urology, Reproduction Center, Yokohama City University Medical Center, Yokohama, 232-0024, Japan; Department of Urology and Renal Transplantation, Yokohama City University Medical Center, Yokohama, 232-0024, Japan; Department of Urology and Renal Transplantation, Yokohama City University Medical Center, Yokohama, 232-0024, Japan; Department of Urology and Renal Transplantation, Yokohama City University Medical Center, Yokohama, 232-0024, Japan; Department of Urology and Renal Transplantation, Yokohama City University Medical Center, Yokohama, 232-0024, Japan; Department of Urology and Renal Transplantation, Yokohama City University Medical Center, Yokohama, 232-0024, Japan; Department of Urology, Yokohama City University Graduate School of Medicine, Yokohama, 236-0004, Japan; Department of Urology and Renal Transplantation, Yokohama City University Medical Center, Yokohama, 232-0024, Japan; Department of Urology, Reproduction Center, Yokohama City University Medical Center, Yokohama, 232-0024, Japan

**Keywords:** case report, ejaculatory disorder, testicular dysgenesis syndrome, mixed gonadal dysgenesis, 45, X/46, XY mosaicism

## Abstract

**Introduction:**

45,X/46,XY mosaicism is a rare anomaly in sexual differentiation, presenting with diverse phenotypes and often leading to infertility due to abnormal gonadal development.

**Aims:**

This report aims to present a case study of a 45,X/46,XY mosaic male patient with an ejaculatory disorder attributed to seminal vesicle dysplasia.

**Methods:**

In this case study, diagnostic procedures encompassed blood tests, semen analysis, chromosomal examination, and imaging studies to assess gonadal morphology. Treatment strategies included attempted varicocelectomy, pharmacological intervention with amoxapine, and surgical testicular sperm extraction. Additionally, the patient underwent assisted reproductive techniques, specifically intracytoplasmic sperm injection (ICSI), to facilitate pregnancy for his wife.

**Results:**

A 32-year-old man could not ejaculate, with post-orgasmic urinalysis revealing minimal sperm presence. Chromosomal analysis confirmed 45,X/46,XY mosaicism. Despite undergoing microsurgical varicocelectomy for clinical varicocele and receiving tricyclic antidepressants, no improvement in semen volume occurred. Imaging studies indicated ejaculatory disorder due to prostate and seminal vesicle aplasia. Consequently, surgical retrieval of testicular sperm was performed, leading to successful pregnancy via ICSI for his wife.

**Conclusion:**

Our approach has effectively addressed ejaculatory disorder in 45,X/46,XY mosaic men, resulting in successful pregnancy.

## Background

45,X/46,XY mosaicism is an uncommon chromosomal anomaly, affecting ~1 in 15 000 live births. It represents a sex differentiation disorder and exhibits various phenotypes, encompassing male, female, and instances where gender determination poses challenges.^1^ Some reports indicate that 90% of cases diagnosed prenatally have a normal male phenotype, whereas those diagnosed postnatally exhibit a wide range of phenotypes.^2^ This condition can lead to a spectrum of gonadal dysgenesis, including infertility, azoospermia, and low testosterone levels.^2^^,^^3^ Herein, we present a case of a 45,X/46,XY mosaic male who presented with an ejaculatory disorder attributed to seminal vesicle dysplasia. He underwent intravesical sperm collection for retrograde ejaculation and left microsurgical low ligation varicocelectomy to better spermatogenesis. Notably, his wife achieved pregnancy through intracytoplasmic sperm injection (ICSI) utilizing surgically retrieved testicular sperm.

## Case presentation

A 32-year-old married man, with a wife of the same age, had a medical history of left cryptorchidism and hypospadias, for which he had undergone orchiopexy and urethroplasty, respectively. There were no notable occurrences in his family history. Moreover, he was unaware of any abnormalities regarding secondary sexual characteristics during puberty. Seeking medical attention due to 1 year and 6 months of infertility, he reported no issues with erections or orgasms since puberty but could not eject semen. A post-orgasmic urinalysis revealed a low sperm count, diagnosed as retrograde ejaculation. Treatment with amoxapine, a tricyclic antidepressant, proved ineffective. Additionally, he was diagnosed with a Grade 2 left-sided varicocele and was subsequently referred to our hospital for further evaluation.

**Figure 1:**
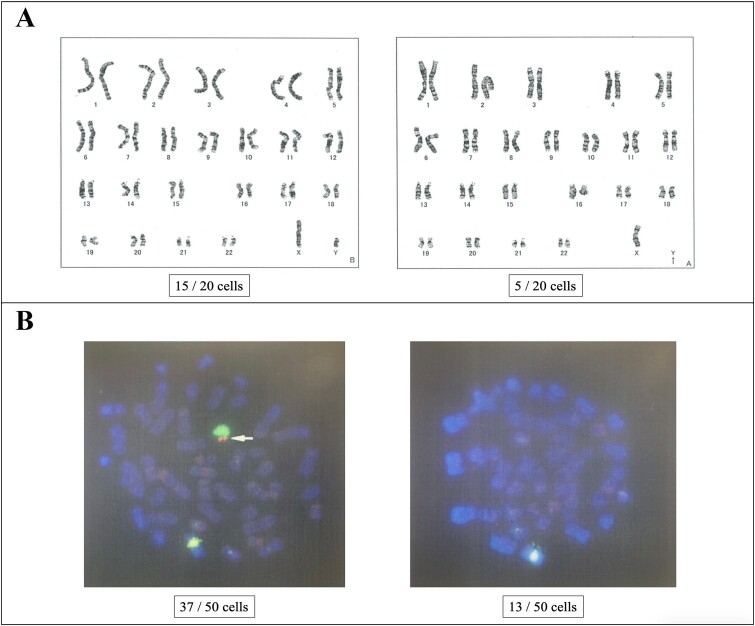
Chromosomal examination. (A) G-banding analysis. In peripheral blood lymphocytes, 45,X was observed in 5 out of 20 cells, while 46,XY was detected in 15 out of 20 cells. (B) Fluorescence in situ hybridization (FISH) analysis. Within peripheral blood lymphocytes, 13 out of 50 cells lacked the sex-determining region Y (SRY), indicating a Y chromosome deficiency.

**Figure 2:**
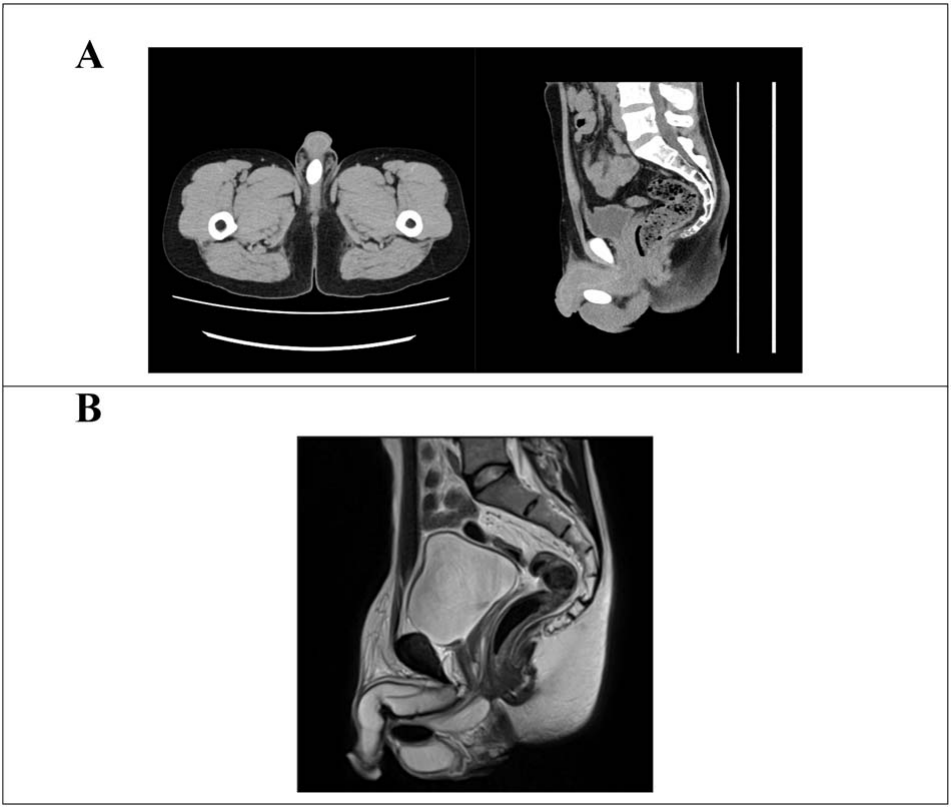
Image examination. (A) Abdominal computed tomography image. Calcification is visible in the midline of the scrotum, positioned just below the penis. (B) Abdominal magnetic resonance imaging scan. The prostate and seminal vesicle glands are not present.

Upon physical examination, the patient measured 154 cm in height, weighed 48 kg, and had a body mass index of 20.2 kg/m^2^. Secondary sexual characteristics were delayed, with pubic hair at Tanner stage IV and phallus at Tanner stage III. Furthermore, orchidometer evaluation revealed bilateral testicular volumes of 6 ml, and the epididymis was normal. The left vas deferens was not palpable, and a Grade 2 left-sided varicocele was observed. An isolated 3-cm-long mass was palpated between the bilateral testes in the scrotum. Furthermore, blood tests revealed the following levels: serum total testosterone 7.05 (1.87–9.02) ng/mL, estradiol 19.0 (15.0–49.0) pg/mL, luteinizing hormone 10.0 (1.7–8.6) mlU/mL, and follicle-stimulating hormone 18.3 (1.6–11.0) mlU/mL. Although a semen analysis was attempted, no ejaculated semen was observed. Post-orgasmic urinalysis after centrifugal separation following retrograde ejaculation showed only one to four sperm in the high-power field. Chromosomal G-banding examination of peripheral blood lymphocytes indicated a karyotype of 45,X/46,XY mosaicism, with 45,X detected in 5 out of 20 cells and 46,XY in 15 out of 20 cells (Figure 1A). Due to the rarity of 45,X/46,XY mosaicism, fluorescence in situ hybridization (FISH) was performed using the sex-determining region Y (SRY) gene of the Y chromosome as a marker to assess the possibility of Y chromosome structural abnormalities. FISH analysis showed no SRY in 13 out of 50 peripheral blood lymphocytes, suggesting a lack of the Y chromosome (Figure 1B). Furthermore, no deletion was detected in the azoospermia factor. Deletions pattern in the azoospermia factor region was examined through polymerase chain reaction-sequence specific oligonucleotide method. Computed tomography revealed a 27-mm-long calcification in the scrotum (Figure 2A), while magnetic resonance imaging displayed aplasia of the prostate and seminal vesicle glands (Figure 2B). Although anejaculation due to impaired semen production associated with seminal vesicle atrophy was considered the primary cause, left microsurgical low ligation varicocelectomy was attempted to improve spermatogenesis. Additionally, the dosage of amoxapine was increased to address the ejaculatory disorder. Intraoperative exploration revealed severe adhesions and deficiency of the left vas deferens following orchiopexy. Despite the increased dosage of amoxapine, no ejaculated semen was observed. However, post-orgasmic urinalysis indicated an improvement in spermatogenesis, with 20–29 sperm seen in the high-power field. Intravesical sperm collection was challenging due to difficulties in urinary catheterization caused by urethral stricture following urethroplasty. Consequently, testicular sperm extraction (TESE) was performed, resulting in the retrieval of a sufficient amount of sperm from right testis. Furthermore, seminiferous tubules were uniform, and mean Johnsen’s score count was 9.0. Histopathological examination revealed no obvious tumor component. Although removal of the calcified scrotal mass during TESE was considered, it was ultimately retained towing to its proximity to the urethra and the lack of symptoms. Subsequently, the patient’s wife achieved pregnancy via ICSI using testicular sperm. In Japan, preimplantation genetic testing for aneuploidy is suggested for repeated miscarriages, and preimplantation genetic testing for structural rearrangement is suggested for chromosomal structural abnormalities. Therefore, this case was not applicable.

## Discussions

45,X/46,XY mosaicism represents a rare chromosomal mutation and is classified as a type of mixed gonadal dysgenesis. Similar conditions involving sex chromosome mosaicism with mixed gonadal dysgenesis includes variations such as 45,X/46,XY,/47,XXY and 45,X/47,XXY. However, it is believed that 45,X/46,XY is more prevalent due to the susceptibility of the abnormal Y chromosome to loss during early somatic cell division, especially when the Y chromosome exhibits morphological abnormalities.^4^ External genitalia exhibit a broad spectrum of phenotypes, ranging from male to intermediate and female, depending on the proportions of 45,X cells to 46,XY cells. Gonadal presentations encompass various combinations, including bilateral testes, unilateral testis with contralateral gonadal or ovarian streaks, and bilateral gonadal streaks.^3^ Individuals with 45,X/46,XY mosaicism have been reported as males, cases with ambiguous gender determination, and females displaying signs of Turner syndrome.^1^ Key clinical features include short stature attributed to deficiency in the short stature homeobox-containing gene and a heightened risk of gonadal tumors.^5^^,^^6^ Timely identification of 45,X/46,XY individuals is crucial, especially when unexplained short stature is evident. As this patient also had an unexplained short stature, we will need to closely monitor the patient’s appearance as well.

Prophylactic gonadectomy is recommended for Turner syndrome cases involving a Y chromosome and for mixed gonadal dysgenesis, where 45,X/46,XY individuals predominate, due to the elevated incidence of gonadoblastoma and dysgerminoma.^6^ Attention should be given to tumor localization during gonadectomy, as tumors may be situated abdominally if not palpable in the scrotum or inguinal region. Tumor development typically occurs around the age of 15, emphasizing the importance of early surveillance. Additionally, the absence of tumor development in 45,XO cases indicates a potential role of the Y chromosome in tumor development.^7^ In this patient, the observed phenotype was male, with a chromosomal examination of peripheral blood indicating a ratio of 25% 45,X and 75% 46,XY. However, chromosomal testing of gonadal tissue was not conducted, leaving the mosaic ratio in the gonads undetermined. Thus, he should be continually monitored for the development of testicular tumors.

The patient’s clinical presentation aligned with features commonly associated with testicular dysgenesis syndrome, including delayed secondary sexual characteristics, impaired spermatogenesis, cryptorchidism, hypospadias, and the presence of a testicular tumor.^8^ Notably, the patient had a history of cryptorchidism and hypospadias. Aplasia of the prostate and seminal vesicle gland, attributed to inadequate gonadal development, was observed, potentially contributing to ejaculatory dysfunction. A previous case report resembling the present one, involving a 45,X/46,XY mosaic individual with ejaculatory disorder and scrotal calcification, indicated accessory genital malformation.^9^ In the present case, short stature, calcification in the scrotum, cryptorchidism, and hypospadias were present, but the cause of ejaculatory dysfunction was unknown and varicocele was absent. Given the potential risk of tumorigenesis associated with calcified masses, diligent long-term monitoring is imperative.

Following successful retrieval of a sufficient quantity of motile sperm through TESE, the patient’s wife underwent oocyte retrieval, controlled ovarian stimulation, and ICSI using testicular sperm, resulting in pregnancy after the fourth blastocyst transfer. While the pregnancy is progressing well, fetal sex determination has not yet been performed in the early stages. Subsequent monitoring of the newborn’s external and internal genitalia is warranted.

In summary, successful management of ejaculatory disorder in 45,X/46,XY mosaic men has facilitated pregnancy achievement.

## List of abbreviations

FISH, fluorescence *in situ* hybridization.

ICSI, intracytoplasmic sperm injection.

SRY, sex-determining region Y.

TESE, testicular sperm extraction.
